# Association Between Spanish-Language Patient Navigation and Adherence to Multi-Target Stool DNA Testing Among Spanish-Speaking Patients at Sanitas Medical Center

**DOI:** 10.1007/s13187-025-02742-8

**Published:** 2025-10-06

**Authors:** Mallik Greene, Timo Pew, Shrey Gohil, A. Burak Ozbay, Juliana Vanessa Rincón López, Paul Limburg, Martha Duarte

**Affiliations:** 1 Exact Sciences Corporation, 5505 Endeavor Ln, Madison, WI 53719 USA; 2 Unisanitas, Bogota, Colombia; 3 Sanitas USA, Keralty Hospital Miami, Miami, FL USA

**Keywords:** Colorectal cancer screening, Mt-sDNA, Patient navigation, Spanish-language outreach, Health disparities

## Abstract

Colorectal cancer (CRC) screening rates remain low among Spanish-speaking populations, in part due to language barriers. This study examined the association between Spanish-language patient navigation on adherence to multi-target stool DNA (mt-sDNA) testing among Spanish-speaking patients at Sanitas Medical Center (SMC). This retrospective analysis utilized data from Exact Sciences Laboratories LLC. It included Spanish-speaking individuals aged 45 to 75 years who received an mt-sDNA test kit between October 1, 2021, and August 1, 2022. Patients were categorized based on Spanish-language outreach (SLO) or English-language outreach (ELO). Adherence was defined as the percentage of kits that were returned with completed tests and successful results within 365 days of the initial shipment date. Chi-square tests and multivariable logistic regression were used to compare adherence between groups and identify factors associated with adherence. The final sample included 3,101 patients in the SLO group and 1,331 in the ELO group. The SLO group showed a 4.4% absolute increase in adherence compared to the ELO group (64.3% vs. 59.9%, *p* = 0.006). At 30 days, adherence was already higher among SLO patients (45.1% vs. 41.5%), and this difference persisted throughout the 12-month follow-up period. The increase in adherence was consistent across subgroups, including patients aged 50–64 years (64.4% vs. 58.5%, *p* = 0.002), males (62.2% vs. 55.5%, *p* = 0.008), and those seen by primary care providers (64.9% vs. 59.8%, *p* = 0.029). In adjusted logistic regression, SLO was associated with 20% higher odds of adherence compared to ELO (OR = 1.20, 95% CI 1.05–1.37, *p* = 0.007). Spanish-language patient navigation was associated with a 4.4% higher adherence to mt-sDNA CRC screening among Spanish-speaking patients. These findings suggest that culturally and linguistically tailored communication may support improved screening participation in underserved populations.

## Introduction

Colorectal cancer (CRC) continues to pose significant health challenges in the United States, marked by its high incidence and mortality rates [1]. CRC remains one of the leading causes of cancer-related deaths despite effective screening strategies that can reduce disease burden through early detection and intervention [1–3]. Nonetheless, disparities in CRC screening uptake persist, particularly among underserved populations such as ethnic and linguistic minorities [4–7].

Among these underserved groups, the Hispanic population in the U.S. stands out as one facing significant barriers to accessing and completing CRC screening [8–10]. A considerable proportion of this population has limited English language proficiency, introducing a significant barrier to effective screening [8, 10]. This linguistic divide complicates health care communication and access, contributing to lower screening rates and increased CRC incidence and mortality within this demographic [7, 8]. In recent years, efforts to address these disparities have increasingly emphasized the importance of culturally and linguistically tailored interventions [8, 11]. One such intervention gaining recognition is Spanish-language patient navigation, a proactive approach that assists individuals in navigating the complexities of the health care system, addressing barriers to care, and facilitating access to appropriate services [12–16].

In the context of CRC screening, Spanish-language patient navigation has been effectively integrated with the multi-target stool DNA test (mt-sDNA), a convenient, non-invasive, at-home screening option [12, 13, 17, 18]. This combination of personalized, Spanish-language patient navigation with mt-sDNA testing offers a promising strategy to improve screening uptake among Spanish-speaking populations.

This study examined the association between Spanish- and English-language patient navigation services on adherence to mt-sDNA testing among Spanish-speaking patients at Sanitas Medical Center (SMC), a Florida-based health care network serving a large population of Spanish-speaking Hispanic patients. The study aimed to highlight the potential value of culturally competent interventions in promoting CRC screening completion by assessing the association between tailored linguistic patient navigation strategies and test adherence.

## Methods

### Data Source

This study utilized aggregated laboratory data from Exact Sciences Laboratories LLC (ESL), the exclusive national laboratory for mt-sDNA testing, on a patient navigation program conducted at the SMC.

### Study Population and Design

This retrospective analysis included Spanish-speaking individuals aged 45 to 75 years old from the SMC who received an mt-sDNA test kit from ESL between October 1, 2021, and August 1, 2022. The individuals were categorized into two groups—Spanish-language outreach (SLO) or English-language outreach (ELO)—based on the language of patient navigation outreach they received after kit shipment. The program was split into 2 phases—the initial outreach phase and the extended outreach phase. During the initial 45 days, the SLO group received outreach in Spanish, and the ELO group received outreach in English. Subsequently, both groups received extended English-language outreach from day 46 until day 365. During the initial 45 days, robust navigation support was provided to individuals in both groups, involving frequent interactions via emails, texts, and/or calls. During the extended English-language outreach period (day 46 onwards), the frequency of these interactions gradually decreased. It is important to note that while the outreach differed by language (Spanish vs. English) during the initial 45-day period, the content, frequency, and format of all messages were standardized between groups. The only tailored component was the use of language-concordant outreach, without additional cultural or content modifications.

The analysis included variables such as patient demographics (age, sex), provider specialty (e.g., primary care provider [PCP] and nurse practitioner/physician assistant [NP/PA]), social vulnerability index (SVI), median household income based on residential ZIP code, and outreach type (SLO or ELO).

The analysis excluded individuals aged below 45 years, those with less than 365 days since the mt-sDNA test kit shipment date, and those who participated in ESL’s clinical studies (to minimize selection bias toward potentially more adherent patients). These data were de-identified and compliant with the Health Insurance Portability and Accountability Act (HIPAA); the current study was exempt from Institutional Review Board (IRB) review.

### Statistical Analysis

Descriptive statistics were used to summarize patient characteristics and 365-day adherence rates. The primary outcome was mt-sDNA test adherence, defined as the percentage of kits returned with completed tests and successful results within 365 days of the initial shipment date. Cumulative adherence over time was assessed and visualized using line plots with 95% confidence intervals at regular time intervals. Between-group differences in adherence were evaluated using χ^2^ or Fisher’s exact tests, as appropriate. Multivariable logistic regression was performed to identify factors independently associated with adherence, including patient navigation outreach language, age, sex, provider specialty, social vulnerability index quartile, and ZIP code–level median household income. All tests were 2-sided, with *P* < 0.05 considered statistically significant.

## Results

### Patient Demographics

The study included 1,331 ELO and 3,101 SLO patients (Table 1). Most patients were aged 50–64 years in both groups (ELO: 70.5%, SLO: 71.8%). No significant age distribution differences were observed (*p *= 0.315). Females represented a slightly higher proportion in both groups (ELO: 57.3%, SLO: 59.0%), but the difference was not statistically significant (*p* = 0.290). The distribution across provider types (PCPs, NPs/PAs, and others) was similar in both groups, with no statistically significant differences (*p* = 0.220). Median household income by ZIP code did not differ significantly between the two groups (*p* = 0.421), with most patients residing in areas with median incomes between $50 and $75 K.

**Table 1:** Patient sociodemographic characteristics by language of outreach

Characteristics	English outreach (*n*, %)	Spanish outreach (*n*, %)	*p*-value
Overall sample	1,331 (100%)	3,101 (100%)	
Age			0.315
45–49	274 (20.6%)	579 (18.7%)	
50–64	938 (70.5%)	2,228 (71.8%)	
65–75	119 (8.9%)	294 (9.5%)	
Sex			0.290
Female	762 (57.3%)	1,830 (59.0%)	
Male	569 (42.7%)	1,271 (41.0%)	
Provider specialty			0.220
NP/PA	634 (47.6%)	1,415 (45.6%)	
Other	68 (5.1%)	194 (6.3%)	
PCP	629 (47.3%)	1,492 (48.1%)	
Social vulnerability index quartile			0.981
1 – Least vulnerable	67 (5.0%)	169 (5.4%)	
2—Less vulnerable	204 (15.3%)	478 (15.4%)	
3—More vulnerable	362 (27.2%)	850 (27.4%)	
4—Most vulnerable	627 (47.1%)	1,441 (46.5%)	
Unknown	71 (5.3%)	163 (5.3%)	
Patient ZIP median household income ($)			0.421
< 50 K	440 (33.1%)	964 (31.1%)	
50–75 K	678 (50.9%)	1,635 (52.7%)	
75 K +	213 (16.0%)	502 (16.2%)	

*NP/PA* nurse practitioner/physician assistant, *PCP* primary care provider

### Adherence to mt-sDNA Testing

Overall, patients in the SLO group had significantly higher adherence (64.3%) compared to those in the ELO group (59.9%; *p* = 0.006) (Table 2). A significant difference in adherence was observed in the 50–64 age group (SLO: 64.4% vs. ELO: 58.5%; *p* = 0.002), while no significant differences were found in the 45–49 or 65–75 age groups. Among males, adherence was significantly higher in the SLO group (62.2% vs. 55.5%; *p* = 0.008), whereas no significant difference was observed among females. Patients in the SLO group also had significantly higher adherence when seen by PCPs (64.9% vs. 59.8%; *p* = 0.029), with no significant differences noted among those seen by non-PCP providers. Across SVI quartiles, adherence was significantly higher among SLO patients in quartiles 3 and 4 (both *p* < 0.05). Additionally, a significant difference was found among patients residing in ZIP codes with a median household income below $50 K (SLO: 65.8% vs. ELO: 57.5%; *p* = 0.004).

**Table 2:** mt-sDNA adherence rates by language of outreach

Characteristics	English outreach (%, [n/N])	Spanish outreach (%, [n/N])	*p*-value
Overall sample	59.9% (797/1,331)	64.3% (1,994/3,101)	0.006
Age			
45–49	59.9% (164/274)	61.0% (353/579)	0.814
50–64	58.5% (549/938)	64.4% (1,435/2,228)	0.002
65–75	70.6% (84/119)	70.1% (206/294)	1.000
Sex			
Female	63.1% (481/762)	65.7% (1,203/1,830)	0.220
Male	55.5% (316/569)	62.2% (791/1,271)	0.008
Provider specialty			
NP/PA	60.3% (382/634)	63.0% (892/1,415)	0.249
Other	57.4% (39/68)	69.1% (134/194)	0.108
PCP	59.8% (376/629)	64.9% (968/1,492)	0.029
Social vulnerability index quartile			
1—Least vulnerable	82.1% (55/67)	58.0% (98/169)	< 0.001
2—Less vulnerable	60.3% (123/204)	62.8% (300/478)	0.602
3—More vulnerable	58.8% (213/362)	67.2% (571/850)	0.007
4—Most vulnerable	57.7% (362/627)	64.0% (922/1,441)	0.008
Unknown	62.0% (44/71)	63.2% (103/163)	0.976
Patient ZIP median household income ($)			
< 50 K	57.5% (253/440)	65.8% (634/964)	0.004
50–75 K	60.5% (410/678)	63.0% (1,030/1,635)	0.274
75 K +	62.9% (134/213)	65.7% (330/502)	0.523

*NP/PA* nurse practitioner/physician assistant, *PCP* primary care provider

### Cumulative Adherence Over Time

**Fig. 1:**
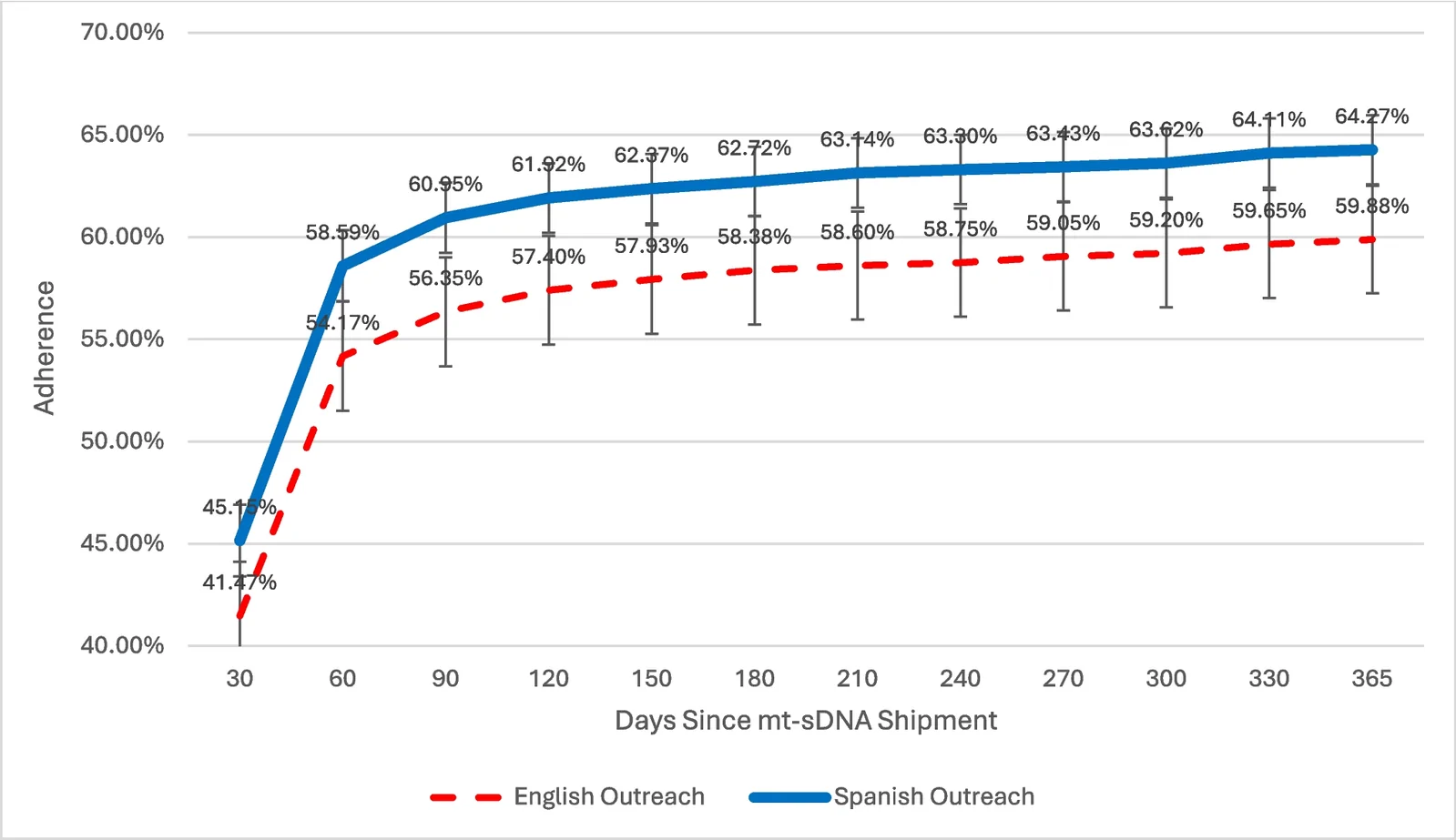
Cumulative adherence to mt-sDNA testing over a 1-year period by language of outreach

Cumulative adherence to mt-sDNA testing was higher among patients who received SLO compared with those who received ELO across the 1-year period (Fig. 1). At 30 days, adherence was 45.1% in the SLO group and 41.5% in the ELO group. This difference persisted over time, with final adherence at 365 days reaching 64.3% for SLO and 59.9% for ELO.

### Factors Associated with Adherence

In the adjusted logistic regression model, SLO was associated with significantly higher odds of adherence to mt-sDNA screening compared to ELO (OR = 1.20, 95% CI 1.05–1.37, *p* = 0.007) (Table 3). Males had lower odds of adherence than females (OR = 0.82, 95% CI 0.73–0.93, *p* = 0.002). Patients aged 65–75 years demonstrated significantly higher odds of adherence compared to those aged 45–49 years (OR = 1.49, 95% CI 1.16–1.92, *p* = 0.002), while the 50–64 age group showed no significant difference (OR = 1.09, 95% CI 0.93–1.27, *p* = 0.302). Provider specialty (PCP vs. NP/PA) and SVI quartile were not significantly associated with adherence. Similarly, the median household income based on ZIP code was not significantly associated with adherence.

**Table 3:** Logistic regression model of factors associated with adherence to mt-sDNA

Variables	Odds ratio	Lower CL	Upper CL	*p*-value
Type of outreach				
English language outreach	Reference	Reference	Reference	
Spanish language outreach	1.199	1.050	1.369	0.007
Sex				
Female	Reference	Reference	Reference	
Male	0.820	0.725	0.928	0.002
Age				
45–49	Reference	Reference	Reference	
50–64	1.085	0.929	1.269	0.302
65–75	1.492	1.158	1.923	0.002
Provider specialty				
NP/PA	Reference	Reference	Reference	
Other	1.125	0.856	1.478	0.398
PCP	1.050	0.925	1.191	0.454
Social vulnerability index quartile				
1—Least vulnerable	Reference	Reference	Reference	
2—Less vulnerable	0.909	0.665	1.242	0.550
3—More vulnerable	1.039	0.769	1.403	0.803
4—Most vulnerable	0.923	0.682	1.249	0.603
Unknown	0.941	0.641	1.380	0.755
Patient ZIP median household income ($)				
< 50 K	Reference	Reference	Reference	
50–75 K	0.926	0.797	1.076	0.315
75 K +	1.013	0.816	1.257	0.906

*CL* confidence limit, *INP/PA* nurse practitioner/physician assistant, *PCP* primary care provider

## Discussion

This study examined the association between Spanish- and English-language patient navigation on mt-sDNA adherence within a Spanish-speaking population served by SMC. It found a significant 4.4% absolute increase in adherence, with the SLO group achieving a rate of 64.3% compared to 59.9% for the ELO group at 365 days. This improvement was consistent across all time periods from 30 to 365 days, likely reflecting the lasting effect of outreach delivered in an individual’s preferred language during the initial 45 days, which may have supported higher adherence even as extended outreach was conducted in English. Such language-concordant communication may enhance patient comprehension and comfort, potentially supporting more informed engagement in the screening process. These findings align with previous research showing that providing healthcare information and support in a person’s preferred or native language improves their understanding, engagement, and adherence to medical recommendations [19, 20]. The observed increase in adherence rates suggests the potential value of patient navigation in overcoming language barriers and reflects the importance of culturally and linguistically tailored interventions.

The adjusted multivariable logistic regression model showed that Spanish-language outreach was associated with 20% higher odds of adherence after accounting for available factors. While not all potential confounders could be assessed, the findings of this model suggest a possible role for culturally and linguistically tailored outreach in supporting screening completion. The model also showed 18% lower odds of adherence among males and 49% higher odds among older patients aged 65–75 years, trends that are generally consistent with prior research, although evidence on sex differences in screening behavior has been mixed across some national studies [21, 22]. The overall adherence rate observed among SLO patients in this study was consistent with previous real-world research reporting comparable adherence among the Hispanic population [23]. While adherence levels were comparable, this study adds evidence that Spanish-language patient navigation may help support patient engagement with the screening process. Providing communication in a patient’s preferred language can improve understanding and confidence, which may encourage the completion of recommended screenings.

The implications of this study may extend beyond the SMC, highlighting a promising approach to improving CRC screening adherence through culturally appropriate assistance, including language support services tailored to diverse populations. Considering the estimated 60 million individuals eligible for CRC screening in the U.S. [24], even a modest 4% incremental increase in adherence, if replicated more broadly, could contribute to meaningful public health improvements by helping to narrow screening gaps and reduce CRC incidence and mortality. While this study focused on Spanish-speaking individuals, the principle of driving effective patient navigation with linguistically tailored outreach has shown promise in other contexts as well [25]. Incorporating culturally and linguistically relevant content into navigation strategies may also complement broader public and patient education efforts aimed at increasing screening uptake. Expanding culturally and linguistically appropriate interventions across more settings and languages could help improve screening adherence and address disparities in CRC and other preventable conditions.

### Limitations

Despite the notable findings, several limitations should be considered when interpreting the results. The retrospective design and reliance on aggregated laboratory data may limit the generalizability of these findings to other health care settings or populations. The focus on mt-sDNA adherence captures only one aspect of CRC screening behavior and does not account for long-term outcomes or other screening modalities. Additionally, the multivariable logistic regression model was limited to a small set of available variables, and potential confounders such as individual socioeconomic factors, health literacy, comorbidities, and healthcare system variables could not be assessed, which may affect the observed associations. While overall adherence differences by SVI quartiles were consistent, one subgroup (SVI quartile 1) with a relatively small sample size showed a different pattern that should be interpreted cautiously. Furthermore, since the extended outreach phase was conducted exclusively in English for all participants, the role of language beyond the initial 45 days could not be isolated, nor could the effects of other cultural or system-level factors. These limitations highlight the need for prospective, more granular studies with diverse populations to validate and expand upon these findings.

## Conclusions

This study demonstrated that Spanish-language patient navigation was associated with a statistically significant 4.4% increase in adherence to mt-sDNA screening among Spanish-speaking patients. The association was consistent across multiple demographic subgroups, highlighting the potential benefit of culturally and linguistically tailored outreach to support CRC screening in underserved populations. While the findings are encouraging, further research is needed to confirm these results across diverse settings and to explore the longer-term impact of such interventions. Nonetheless, broader implementation of linguistically appropriate patient navigation may contribute meaningfully to reducing disparities in preventive care and improving screening adherence.

## Data Availability

Study data are available upon reasonable request, subject to applicable regulations and company policies.

## Code Availability

Study code may be made available upon reasonable request, in accordance with the company’s protocols and confidentiality requirements.
